# Strategies for the development and approval of COVID-19 vaccines and therapeutics in the post-pandemic period

**DOI:** 10.1038/s41392-023-01724-w

**Published:** 2023-12-21

**Authors:** Danyi Ao, Xuemei He, Jian Liu, Li Xu

**Affiliations:** State Key Laboratory of Biotherapy, West China Hospital, Sichuan University, 610041 Sichuan, People’s Republic of China

**Keywords:** Infectious diseases, Infectious diseases

## Abstract

The spread of severe acute respiratory syndrome coronavirus 2 (SARS-CoV-2) has resulted in significant casualties and put immense strain on public health systems worldwide, leading to economic recession and social unrest. In response, various prevention and control strategies have been implemented globally, including vaccine and drug development and the promotion of preventive measures. Implementing these strategies has effectively curbed the transmission of the virus, reduced infection rates, and gradually restored normal social and economic activities. However, the mutations of SARS-CoV-2 have led to inevitable infections and reinfections, and the number of deaths continues to rise. Therefore, there is still a need to improve existing prevention and control strategies, mainly focusing on developing novel vaccines and drugs, expediting medical authorization processes, and keeping epidemic surveillance. These measures are crucial to combat the Coronavirus disease (COVID-19) pandemic and achieve sustained, long-term prevention, management, and disease control. Here, we summarized the characteristics of existing COVID-19 vaccines and drugs and suggested potential future directions for their development. Furthermore, we discussed the COVID-19-related policies implemented over the past years and presented some strategies for the future.

## Introduction

As of May 5, 2023, with the decrease in global mortality rates, hospitalizations, and severe cases caused by the severe acute respiratory syndrome coronavirus 2 (SARS-CoV-2), the World Health Organization (WHO) announced that Coronavirus disease 2019 (COVID-19) no longer considered a Public Health Emergency of International Concern.^1^ Similarly, on May 11, 2023, the United States government terminated the COVID-19 Public Health Emergency.^2^ More and more countries have decided to transition from emergency response to long-term management. However, it is crucial not to underestimate the ongoing pandemic.

Until June 14, 2023, over 130 billion vaccine doses had been administered globally, and approximately 70% of the total population has received at least one COVID-19 vaccine dose.^3,4^ Despite vaccination efforts, new infections and thousands of deaths are reported weekly. Even among the vaccinated population, there remains a notable incidence of breakthrough infections with new variants, resulting in a persistent strain on health systems.^5,6^ Furthermore, vaccines and drugs against COVID-19 have observed a marked decline in effectiveness against SARS-CoV-2 variants.^7^ These findings raise concerns about the efficacy of current vaccines and drugs. The efficacy of preventive and therapeutic measures is influenced by many factors, among which the mutations of the key structural domains in the virus play a decisive role. Ongoing mutations may confer variants with heightened transmissibility and immune escape capabilities, thereby diminishing the protective capabilities of vaccines and drugs. Presently, novel SARS-CoV-2 variants continue to emerge. For instance, WHO has recently classified EG.5 and XBB.1.9.1 as “variants of interest” and “variants under monitoring”, respectively.^8^ EG.5 variant was first reported on 17 February 2023 and rapidly supplanted other strains, leading to a surge in infections in some countries.^9^ The evolution results of SARS-CoV-2 have shown that each variant of concern (VOC) has evolved independently from a previously circulating ancestor during the pandemic, which indicates that SARS-CoV-2 variants follow various mutational paths to develop adaptations for human hosts. Hence, the SARS-CoV-2 virus may evolve into variants with increased transmissibility and greater immune evasion potential than those existing variants of concern, consequently damaging the efficacy of current vaccines and drugs.

When exposure to the constantly mutating SARS-CoV-2 virus is inevitable, countries must fortify their response measures, including consistently surveilling prevalent variants, developing next-generation medicines, and obtaining appropriate authorization. These proactive actions are vital in adequately preparing for potential threats from COVID-19 and future pandemics.

## Current COVID-19 preventive and therapeutic strategies

### Current vaccines against COVID-19

**Fig. 1:**
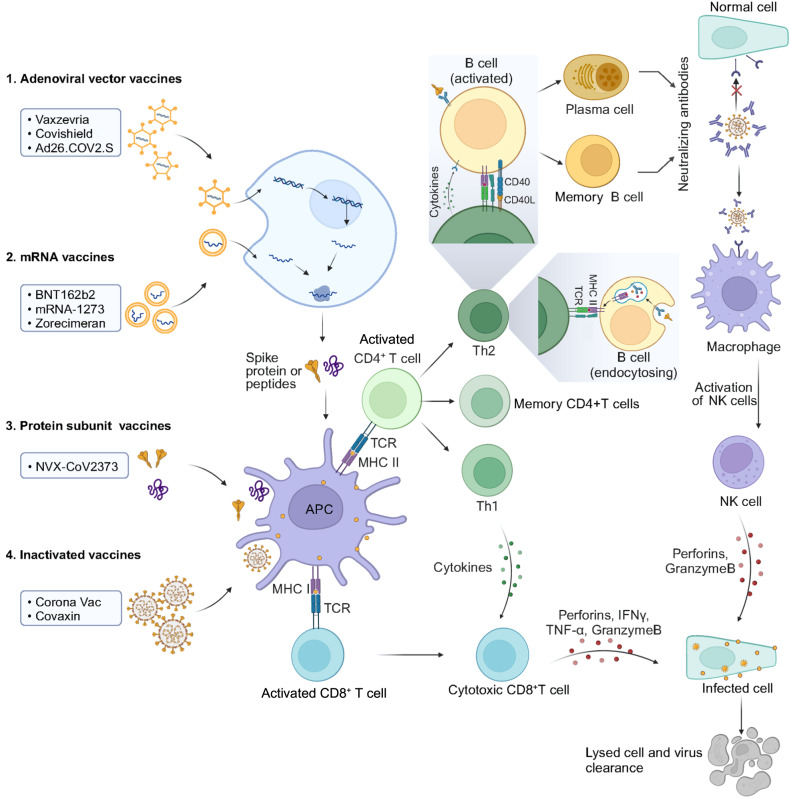
Molecular mechanisms of different types of COVID-19 vaccines. After administration, COVID-19 vaccines would elicit cellular and humoral immune responses directed against the SARS-CoV-2 virus. The antigens that translate by adenovirus vector and mRNA vaccines or contain within protein and inactivated vaccines are identified by antigen-presenting cells (APCs) and subsequently presented to T cells, thereby initiating T cell activation. Cytokines generated by Th1 cells serve to stimulate CD8^+^ T cells, inducing the production of perforin, ultimately resulting in the demise of infected cells. Th2 cells activate B cells, prompting the generation of memory B cells and plasma B cells. Plasma B cells produce specific neutralizing antibodies aimed at eliminating the virus

Vaccination is essential in the initial stages of COVID-19 prevention. As of August 8, 2023, more than 58 vaccines have been approved by WHO (Table 1), with more vaccines in clinical trials (Table 2).^10–29^ These vaccines are produced by various platforms, encompassing inactivated, protein subunit, mRNA, and adenovirus vector vaccines (Fig. 1).

**Table 1:** The summary of currently WHO-approved/PQ evaluating COVID-19 vaccines

Type	Platform	Manufacture/WHO EUL holder	Name of vaccine	Virus strain	Dosage	References
mRNA	Nucleoside modified mRNA	Pfizer-BioNTech	BNT162b2/ Comirnaty Tozinameran (INN)	Wild-type	30 µg RNA (2×)	^10^
			Comirnaty COVID-19 (Bivalent)	BA.4, BA.5	30 µg RNA (2×)	^11^
			Comirnaty Bivalent Original/Omicron BA.1	Wild-type, BA.1	N/A	^12^
	mRNA-based vaccine encapsulated in lipid nanoparticle (LNP)	Moderna Biotech	mRNA-1273	Wild-type	100 µg RNA (2×)	^13^
			mRNA-1273.214	Wild-type, BA.1	100 µg RNA (2×)	^14,15^
			mRNA-1273.222	Wild-type, BA.4/5	100 µg RNA (2×)	^16^
		CureVac	Zorecimeran (INN)	Wild-type	N/A	^13^
	RNA vaccine	Arcturus Therapeutics	ARCT-154	Wild-type	5 µg RNA (2×)	^17^
		Gennova Biopharmaceuticals Limited	GEMCOVAC-19	Wild-type	N/A	^12^
Adenovector	Recombinant ChAdOx1 adenoviral vector encoding the Spike protein	AstraZeneca, AB	ChAdOx1 (AZD1222)	Wild-type	5 × 10^10^ adenovirus vector particles (2×)	^18^
		R-PHARM	Vaccine R-COVI		N/A	^13^
	Recombinant, replication-incompetent adenovirus type 26 (Ad26) vectored vaccine encoding the Spike protein	Janssen–Cilag International NV	Ad26.COV2.S	Wild-type	5 × 10^10^ adenovirus vector particles (1×)	^19^
	Recombinant Novel Coronavirus Vaccine (Adenovirus Type 5 Vector)	CanSino Biological Inc.	Ad5-nCoV/Convidecia	Wild-type	5 × 10^10^ adenovirus vector particles (2×)	^20^
		CanSino Biological Inc.	Convidecia Air^TM^	Wild-type	1 × 10^10^ adenovirus vector particles (2×)	^13^
	Human Adenovirus rAd26 and rAd5 Vector-based Covid-19 vaccine	Gamaleya	Sputnik V	Wild-type	10 × 10^10^ adenovirus vector particles (2×)	^21^
Inactivated	Inactivated, produced in Vero cells	Beijing Institute of Biological Products Co., Ltd. (BIBP)	SARS-CoV-2 Vaccine (Vero Cell), Inactivated (lnCoV)	Wild-type	N/A	^13^
		Sinovac Life Sciences Co., Ltd.	Coronavac^TM^	Wild-type	3 µg proposed (2×)	^13^
		Bharat Biotech, India	iNCOVACC	Wild-type	N/A	^12^
		Chumakov Center	KoviVac	Wild-type	N/A	^12^
		Health Institutes of Turkey	Turkovac	Wild-type	N/A	^12^
		Organization of Defensive Innovation and Research	FAKHRAVAC (MIVAC)	Wild-type	N/A	^12^
		Shenzhen Kangtai Biological Products Co	KCONVAC	Wild-type	N/A	^12^
		Shifa Pharmed Industrial Co	COVIran Barekat	Wild-type	N/A	^12^
		Sinopharm (Beijing)	Covilo	Wild-type	N/A	^12,22^
		Research Institute for Biological Safety Problems (RIBSP)	QazVac	Wild-type	N/A	^13^
		Valneva	VLA2001	Wild-type	N/A	^13^
		Sinopharm / WIBP	Inactivated SARS-CoV-2 Vaccine (Vero Cell)	Wild-type	N/A	^13^
		Shifa Pharmed - Barkat	CovIran® vaccine	Wild-type	N/A	^13^
	Whole-Virion Inactivated Vero Cell	Bharat Biotech, India	COVAXIN	Wild-type	6 µg proposed (2×)	^13^
Subunit	Recombinant nanoparticle prefusion spike protein formulated with Matrix-M™ adjuvant	Novavax, Inc.	NVX-CoV2373/Covovax	Wild-type	5 µg S (+50 µg adjuvant) (2×)	^13^
		SK Bioscience	Nuvaxovid	Wild-type	5 µg S (+50 µg adjuvant) (2×)	^13^
	Recombinant protein subunit	SK Bioscience	GBP510	Wild-type	N/A	^13^
		Sanofi	CoV2 preS dTM-AS03 vaccine	Wild-type	N/A	^23^
		Zhifei Longcom, China	Recombinant Novel Coronavirus Vaccine (CHO Cell)	Wild-type	25 µg NCP-RBD (+adjuvant) (3×)	^13^
		Sanofi/GSK	VidPrevtyn Beta	Beta	N/A	^13^
		Nanogen	Nanocovax	Wild-type	N/A	^24^
		Cinnagen	SpikoGen	Wild-type	N/A	^13^
		Sinocelltech, Ltd	SCTV01C	Alpha, Beta	N/A	^13^
		Medigen	MVC-COV1901	Wild-type	N/A	^13^
		Center for Genetic Engineering and Biotechnology (CIGB)	Abdala	Wild-type	50 mcg RBD + 0.30 mg aluminum hydroxide	^25^
		Razi Vaccine & Serum Research Institute	Razi Cov Pars Vaccine	Wild-type	N/A	^13^
		WestVac Biopharma	Recombinant COVID-19 Vaccine	Wild-type	N/A	^13^
		Stelis Biopharma Limited	AKS-452 Vaccine (AmbiVax-C^TM^)	Wild-type	two 45 μg doses or a single 90 μg dose	^26^
		PT Biofarma	SARS CoV-2 RBD	Wild-type	N/A	^13^
		Bagheiat-allah University of Medical Sciences	Noora vaccine	Wild-type	80 μg of recombinant RBD protein (+adjuvant) (2×)	^12,27^
		Instituto Finlay de Vacunas Cuba	Soberana 02	Wild-type	50 µg RBD	^12^
		Instituto Finlay de Vacunas Cuba	Soberana Plus/ FINLAY-FR-1A	Wild-type	50 µg RBD	^12^
		National Vaccine and Serum Institute	Recombinant SARS-CoV-2 Vaccine (CHO Cell)	Wild-type	N/A	^12^
		Livzon Mabpharm Inc	V-01	Wild-type	10 μg of fusion protein (+adjuvant) (2×)	^12,28^
		SK Bioscience	SK Bio-SKYCovione	Wild-type	N/A	^12^
		Biological E	Corbevax	Wild-type	N/A	^13^
		HIPRA	BIMERVAX	Alpha, Beta	N/A	
		Liaoning Yisheng Biopharma Co.	PIKA recombinant protein	N/A	N/A	^13^
	Plant-based virus-like particle (VLP), recombinant protein	Medicago	COVIFENZ®	Wild-type	N/A	^13^
	Modified recombinant spike protein	Shionogi & Co., Ltd	S-268019	Wild-type	N/A	^13^
	Recombinant SARS-CoV-2 Spike-Trimer fusion protein	Clover Biopharmaceuticals	SCB-2019	Wild-type	30 μg S (+adjuvant) (2×)	^29^
	Protein-peptide vaccine	Vaxxinity	UB-612	Wild-type	N/A	^13^

*EUL* emergency use listing procedure, *N/A* not available

**Table 2:** The summary of COVID-19 vaccines in clinical study

Platform	Type of candidate vaccine	Developers	Number of doses	Route of administration
RNA-based vaccine	RVM-V001	RVAC Medicines	1	IM
	LNP-nCoVsaRNA	Imperial College London	2	IM
	SARS-CoV-2 mRNA vaccine (ARCoV)	Academy of Military Science (AMS), Walvax Biotechnology and Suzhou Abogen Biosciences	2	IM
	ChulaCov19 mRNA vaccine	Chulalongkorn University	2	IM
	PTX-COVID19-B, mRNA vaccine	Providence Therapeutics	2	IM
	CoV2 SAM (LNP) vaccine. A self-amplifying mRNA (SAM) lipid nanoparticle (LNP) platform + Spike antigen	GlaxoSmithKline	2	IM
	mRNA-1273.351	Moderna + National Institute of Allergy and Infectious Diseases (NIAID)	3	IM
	mRNA-1273.529-Booster	ModernaTX, Inc.	1	IM
	MV-014-212	Meissa Vaccines, Inc.	1	IN
	DS-5670a, coronavirus-modified uridine RNA vaccine (SARS-CoV-2)	Daiichi Sankyo Co., Ltd.	2	IM
	HDT-301	SENAI CIMATEC	2	IM
	mRNA COVID-19 vaccine (SW-BIC-213)	Shanghai East Hospital and Stemirna Therapeutics	2	IM
	LNP-nCOV saRNA-02 vaccine	MRC/UVRI and LSHTM Uganda Research Unit	2	IM
	ARCT-165 mRNA Vaccine	Arcturus Therapeutics, Inc.	2	IM
	ARCT-021 mRNA Vaccine	IM Arcturus Therapeutics, Inc.	2	IM
	HDT-301 vaccine	HDT Bio	1-2	IM
	VLPCOV-01, self-amplifying RNA vaccine against the coronavirus	VLP Therapeutics Japan GK	2	IM
	EG-COVID vaccine	EyeGene Inc.	3	IM
	Coronavirus mRNA vaccine (LVRNA009)	AIM Vaccine and Liverna Therapeutics	2	IM
	CV2CoV, mRNA vaccine	CureVac AG	1	IM
	mRNA vaccine (MIPSCo-mRNA-RBD-1)	University of Melbourne	1	IM
	COVID-19 mRNA Vaccine (SYS6006)	CSPC ZhongQi Pharmaceutical Technology Co., Ltd.	2	IM
	Lyophilized COVID-19 mRNA Vaccine	Wuhan Recogen Biotechnology Co., Ltd.	1	IM
	A self-amplifying RNA (saRNA) boost vaccines (AAHI-SC2 and AAHI-SC3)	ImmunityBio, Inc.	1	IM
	mRNA-1073; (COVID-19/Influenza) Vaccine	Moderna TX.	2	IM
	ABO1009-DP (COVID-19 Omicron) mRNA Vaccine	Suzhou Abogen Biosciences Co., Ltd.	1	IM
	Investigational CV0501 mRNA COVID-19 Vaccine	GlaxoSmithKline	1	IM
	GLB-COV2-043, an mRNA booster vaccine candidate	GreenLight Biosciences, Inc.	1	IM
	JCXH-221, an mRNA-based	Immorna Biotherapeutics, Inc.	1	IM
	mRNA-based COVID-19 vaccine (COReNAPCIN)	ReNAP Technology	1	IM
DNA-based vaccine	INO-4800+electroporation	Inovio Pharmaceuticals + International Vaccine Institute + Advaccine (Suzhou) Biopharmaceutical Co., Ltd	2	ID
	AG0301-COVID19	AnGes + Takara Bio + Osaka University	2	IM
	GX-19N	Genexine Consortium	2	IM
	Covigenix VAX-001 - DNA vaccines + proteo-lipid vehicle (PLV) formulation	Entos Pharmaceuticals Inc.	2	IM
	CORVax12 - Spike (S) Protein Plasmid DNA Vaccine	OncoSec Immunotherapies; Providence Health & Services	2	ID
	bacTRL-Spike oral DNA vaccine	Symvivo Corporation	1	Oral
	GLS-5310	GeneOne Life Science, Inc.	2	ID
	COVIGEN	University of Sydney, Bionet Co., Ltd; Technovalia	2	ID, IM
	COVID-eVax, a candidate plasmid DNA vaccine of the Spike protein	Takis + Rottapharm Biotech	2	IM, IM + electroporation
	SC-Ad6-1, Adneviral vector vaccine	Tetherex Pharmaceuticals Corporation	1-2	IM
	AG0302-COVID19	AnGes, Inc/Osaka University	2-3	IM
	Plasmid DNA vaccine SCOV1 + SCOV2. COVIDITY	Scancell Ltd	2	ID
	VB10.2129, a DNA plasmid vaccine encoding the receptor-binding domain (RBD)	Vaccibody AS	1-2	IM
	VB10.2210, DNA plasmid vaccine, encodes multiple immunogenic and conserved T cell epitopes spanning multiple antigens across the SARS-CoV-2 genome	Vaccibody AS	1-2	IM
	SARS-CoV-2 DNA vaccine (delivered IM followed by electroporation)	The University of Hong Kong; Immuno Cure 3 Limited	2	IM
	Prophylactic pDNA Vaccine Candidate Against COVID-19	Imam Abdulrahman Bin Faisal University	3	IM
	Booster DNA vaccine delivered by in vivo “EPS Gun” from IGEA optimized for Electro Gene Transfer (EGT) vaccination	Matti Sällberg, Karolinska Institutet	1	IM
Virus-like particle	RBD SARS-CoV-2 HBsAg VLP vaccine	Serum Institute of India + Accelagen Pty + SpyBiotech	2	IM
	VBI-2902a	VBI Vaccines Inc.	2	IM
	SARS-CoV-2 VLP Vaccine	The Scientific and Technological Research Council of Turkey	2	SC
	ABNCoV2 capsid virus-like particle (cVLP) +/− adjuvant MF59	Radboud University	2	IM
	SARS-CoV-2 Vaccine LYB001, a receptor-binding domain (RBD) from SARS-CoV-2 and virus-like particle (VLP) vector, adjuvanted with aluminum hydroxide	Yantai Patronus Biotech Co., Ltd.	3	IM
	VBI-2901e. The trivalent vaccine composed of virus-like particles (eVLPs) to express the spike proteins of three coronaviruses: SARS-CoV-2, SARS-CoV-1 and MERS-CoV, with aluminum phosphate and E6020 adjuvants	VBI Vaccines Inc.	2	IM
Viral vector (Replicating)	DelNS1-2019-nCoV-RBD-OPT1 (Intranasal flu-based-RBD)	University of Hong Kong, Xiamen University and Beijing Wantai Biological Pharmacy	2	IN
	rVSV-SARS-CoV-2-S Vaccine (IIBR-100)	Israel Institute for Biological Research	1	IM
	COVIVAC	Institute of Vaccines and Medical Biologicals, Vietnam	2	IM
	NDV-HXP-S; A Live Recombinant Newcastle Disease Virus-vectored COVID-19 Vaccine	Sean Liu, Icahn School of Medicine at Mount Sinai	1	IN
Viral vector (Replicating) + APC	Dendritic cell vaccine AV-COVID-19. A vaccine consisting of autologous dendritic cells loaded with antigens from SARS-CoV-2, with or without GM-CSF	Aivita Biomedical, Inc; National Institute of Health Research and Development; Ministry of Health Republic of Indonesia	1	IM
	Covid-19/aAPC vaccine. The Covid-19/aAPC vaccine is prepared by applying lentivirus modification with immune modulatory genes and the viral minigenes to the artificial antigen-presenting cells (aAPCs).	Shenzhen Geno-Immune Medical Institute	3	SC
Viral vector (Non-replicating) + APC	LV-SMENP-DC vaccine. Dendritic cells are modified with lentivirus vectors expressing Covid-19 minigene; SMENP and immune modulatory genes. CTLs are activated by LV-DC presenting COVID-19-specific antigens.	Shenzhen Geno-Immune Medical Institute	1	SC & IV
Viral vector (Non-replicating)	GRAd-COV2 (Replication defective Simian Adenovirus (GRAd) encoding S)	ReiThera + Leukocare + Univercells	2	IM
	VXA-CoV2-1 Ad5 adjuvanted Oral Vaccine platform	Vaxart	2	Oral
	MVA-SARS-2-S	University of Munich (Ludwig-Maximilians)	2	IM
	Human Adenovirus Type 5: hAd5 S+N bivalent vaccine (S-Fusion + N-ETSD). E2b- Deleted Adeno	ImmunityBio, Inc	1-2	SC
	COH04S1 (MVA-SARS-2-S) - Modified vaccinia ankara (sMVA) platform + synthetic SARS-CoV-2	City of Hope Medical Center + National Cancer Institute	1-2	IM
	AdCLD-CoV19 (adenovirus vector) AdCLD-CoV19-1 OMI	Cellid Co., Ltd.	1	IM
	BBV154, Adenoviral vector COVID-19 vaccine	Bharat Biotech International Limited	1	IN
	Chimpanzee Adenovirus serotype 68 (ChAd) and self-amplifying mRNA (SAM) vectors expressing spike alone or spike plus additional SARS-CoV-2 T cell epitopes.	Gritstone Oncology	2-3	IM
	PIV5 vector that encodes the SARS-CoV-2 spike protein	CyanVac LLC	1	IN
	AZD2816; adenoviral vector ChAdOx platform and based on the Beta (B.1.351) variant	AstraZeneca + University of Oxford	2	IM
	AAV5-RBD-S vaccine (BCD-250), A recombinant Adenovirus-Associated viral Vector (AAV-5) encoding spike protein	Biocad	1	IM
	Ad5-triCoV/Mac or ChAd-triCoV/Mac, new experimental adenovirus-based vaccines expressing SARS-CoV-2 spike, nucleocapsid and RNA polymerase proteins	McMaster University	1	AE
	Ad26.cov2.s+bcg vaccine. AD26-BCG	Han Xu, M.D., Ph.D., FAPCR, Sponsor-Investigator, IRB Chai	1	ID
	MVA-SARS-2-ST Vaccine	Hannover Medical School	1	IH
	CoVacHGMix adenoviral vector vaccine	Ankara City Hospital Bilkent	2	IM
	Recombinant COVID-19 Vaccine (Adenovirus Vector)	Wuhan BravoVax	1	IN
Protein subunit	KBP-COVID-19 (RBD-based)	Kentucky Bioprocessing Inc.	2	IM
	IMP CoVac-1 (SARS-CoV-2 HLA-DR peptides)	University Hospital Tuebingen	1	SC
	Baiya SARS-CoV-2 VAX1, a plant-based subunit vaccine (RBD-Fc + adjuvant)	Baiya Phytopharm Co., Ltd.	2	IM
	Recombinant COVID-19 Variant Vaccine (Sf9 Cell)	Westwac Biopharma Co., Ltde	1	IM
	Recombinant COVID-19 Trivalent (XBB.1.5 + BA.5+Delta) Protein Vaccine (Sf9 Cell)	Westwac Biopharma Co., Ltde	1	IM
	Recombinant COVID-19 Bivalent (XBB.1.5+Prototwpe) Protein Vaccine (Sf9 Cell)	Westwac Biopharma Co., Ltde	1	IM
	COVAC-1 and COVAC-2 subunit vaccine (spike protein) + SWE adjuvant	University of Saskatchewan	2	IM
	MF59 adjuvanted SARS-CoV-2 Sclamp vaccine	The University of Queensland	2	IM
	spike ferritin nanoparticle	Walter Reed Army Institute of Research (WRAIR)	2-3	IM
	EuCorVac-19	POP Biotechnologies and EuBiologics Co., Ltd	2	IM
	ReCOV	Jiangsu Rec-Biotechnology	2	IM
	CoVepiT vaccine	OSE Immunotherapeutics	1-2	SC
	OGEN1, protein-based vaccine	USSF/Vaxform	1-2	Oral
	RBD protein recombinant SARS-CoV-2 vaccine (Noora Vaccine)	Bagheiat-allah University of Medical Sciences/AmitisGen	3	IM
	SCB-2020S, an adjuvanted recombinant SARS-CoV-2 trimeric S-protein (from B.1.351 variant)	Clover Biopharmaceuticals AUS Pty Ltd	2	IM
	202-CoV; SARS-CoV-2 spike trimer protein + adjuvant CpG7909	Shanghai Zerun Biotechnology + Walvax Biotechnology + CEPI	2	IM
	Recombinant protein RBD fusion dimer adjuvanted vaccine (COVID-19 Vaccine Hipra) PHH-1V	Laboratorios Hipra, S.A.	2	IM
	Versamune-CoV-2FC vaccine, recombinant S1 antigen	Farmacore Biotecnologia Ltda	3	N/A
	SII B.1.351 + Matrix-M1 adjuvant, a monovalent SII SARS-CoV-2 B.1.351 (Beta) variant vaccine	Novavax	2	IM
	SII Bivalent + Matrix-M1 adjuvant, a bivalent SII vaccine containing antigen for both the ancestral strain and B.1.351 (Beta) variant of SARS-CoV-2	Novavax	1	IM
	SII B.1.617.2 + Matrix-M1 adjuvant, a monovalent SII SARS-CoV-2 B.1.617.2 (Delta) variant vaccine	Novavax	1-2	IM
	PepGNP-SARSCoV2, A CD8 T-cell priming adaptive vaccine composed of a Coronavirus-specific peptides mounted on a gold nanoparticle	Emergex Vaccines Holding Limited	2	ID
	SARS-CoV-2 Vaccine (IN-B009)	HK inno.N Corporation	2	IM
	Adjuvanted SARS-CoV-2 (COVID-19) Beta Variant RBD Recombinant Protein (DoCo-Pro-RBD-1+MF59)	University of Melbourne	1	IM
	Betuvax-CoV-2 COVID-19 vaccine	Human Stem Cell Institute, Russia	2	IM
	VXS-1223U Microarray patch (HD-MAP) vaccine composed of ARS-CoV-2 spike protein (HexaPro)	Vaxxas Pty Ltd.	1	ID
	Recombinant SARS-CoV-2 S-Trimer Vaccine (CHO Cell) booster	Binhui Biopharmaceutical Co., Ltd.	1	IM
Live attenuated virus	COVI-VAC	Codagenix/Serum Institute of India	1-2	IN
	MV-014-212, a live attenuated vaccine that expresses the spike (S) protein of SARS-CoV-2	Meissa Vaccines, Inc.	1	IN
Inactivated Virus	Koçak-19 Inactivated adjuvant COVID-19 viral vaccine	Kocak Farma	2	IM
	Adjuvanted inactivated vaccine against SARS-CoV-2	The Scientific and Technological Research Council of Turkey (TÜBITAK)	2	SC
	Covi Vax, inactivated coronavirus vaccine	National Research Centre, Egypt	2	IM
	Osvid-19 inactivated vaccine for Covid-19	Osve Pharmaceutical Company	2	IM
	EgyVax Inactivated SARS-CoV-2 vaccine candidate	Eva Pharma	2	IM
	UNAIR Inactivated COVID-19 Vaccine	Airlangga University, Indonesia	2	IM
	Omicron COVID-19 inactivated Vaccine (Vero Cell)	China National Biotec Group Company Limited	2	IM
	Inactivated COVID-19 vaccine	KM Biologics Co., Ltd.	2	IM

Data were collected from WHO

*SC* subcutaneous, *ID* intradermal, *IM* intramuscular, *IN* intranasal, *AE* aerosol, *IH* inhaled, *N/A* not available

mRNA vaccines revolutionized vaccine development, offering a faster production speed for COVID-19 vaccines. Moreover, mRNA vaccines demonstrate favorable safety profiles attributed to their non-integrating and non-infectious characteristics.^30^ WHO has approved several mRNA vaccines, such as BNT162b2.^13^ More vaccines against new variants of SARS-CoV-2 are currently being tested in clinical trials.^31^ However, the development of mRNA vaccines also faces several challenges, such as effective delivery systems, susceptibility to degradation, and stringent temperature requirements during transportation and storage.^32^ In addition, the high cost of production expenses also limits the widespread use of mRNA vaccines in low- and middle-income nations.

According to Table 1, more than 22 protein subunit vaccines have received market approval. Protein subunit vaccine has been utilized for decades, exhibiting a high degree of stability during both storage and transportation. Protein subunit vaccines are the most extensively researched and approved type of SARS-CoV-2 vaccines. Currently, there are many production routes for protein vaccines, such as insect and plant cell expression systems. Insect cell expression system can produce large eukaryotic proteins at high levels. This system also has significant advantages in enhanced protein stability and vaccine safety.^33–38^ Plant-based expression system also provides a high level of safety and can facilitate regulatory approvals without costly infrastructure.^39,40^ Proteins expressed from the chloroplast genome can retain their structure and function at room temperature, enabling long-term storage in non-refrigerated environments.^41^ Compared with inactivated viruses, subunit protein vaccines use specific immunogenic epitopes, eliciting more robust immune responses and neutralizing antibodies.^42^ COVID-19 protein subunit vaccines can be categorized into two main types: S and receptor-binding domain (RBD) protein-based vaccines.^43^ NVX-CoV2373 is the initial vaccine authorized by the European Medicines Agency (EMA) based on the S protein subunit.^44^ Two doses of NVX-CoV2373 delivered 89.7% protection against infection and displayed robust effectiveness against the B.1.1.7 variant during the Phase III clinical trial.^45^ Challenges for protein subunit vaccines include antigen selection and maintaining durable immune responses, necessitating the use of proper adjuvants.^46^

Adenovirus vector vaccines utilize replication-incompetent engineered viruses that carry genetic material encoding proteins. One notable advantage of adenovirus vector vaccines is their capacity to elicit long-lasting immunity with only one or two doses.^47^ There are several approved adenovirus vector vaccines, including Convidecia, Vaxzevria, Covishield, and Ad26.COV2.S.^13^ Moreover, the intranasal administration of adenovirus vector vaccines leverages their mucosal tropism to create a better immune microenvironment in the nasal mucosa, effectively preventing respiratory virus invasion. However, compared to inactivated or protein subunit vaccines, adenovirus vector vaccines pose a heightened risk of complications, particularly thrombocytopenia.^48^

Inactivated vaccines are a well-established platform with a long history, recognized for their relatively straightforward production process, which facilitates rapid and large-scale manufacturing. Inactivated vaccines, such as CoronaVac, employ the complete virus as an immunogen, stimulating a wider range of antibodies that target various epitopes.^43,48,49^ Nevertheless, compared to other types of vaccine, inactivated vaccines may have comparatively modest immunogenicity. For instance, a previous investigation revealed that patients inoculated with the Pfizer mRNA vaccine exhibited significantly higher levels of neutralizing antibodies than those vaccinated with inactivated vaccines.^50^

Although there are multiple COVID-19 vaccines on the market, and under preclinical or clinical (Tables 1 and 2), the emergence of the Omicron subvariants, especially XBB.1.5, significantly compromised the effectiveness of most current vaccines.^7^ For example, a study conducted in China gathered serum samples from healthy volunteers 14 days after receiving three doses of CoronaVac. These samples were then assessed for their neutralizing capabilities against various SARS-CoV-2 variants.^51^ Results illustrated that vaccinating CoronaVac as a booster maintained a detectable neutralizing ability for WT. However, partial neutralization ability was lost for descendants of BA.2, especially XBB.1.5, which showed about 7-fold reductions compared to WT.^51^ In addition, the virus seems to evolve faster than vaccine development. For instance, while many pharmaceutical companies were scrambling to develop vaccines against XBB.1.5, the CDC reported that the proportion of EG.5 and FL.1.5.1 amounted to 33.8%, surpassing the proportion of XBB.1.5 by December 14, 2023. Moreover, CDC predicted that before November 11, 2023, another new variant, HV.1 would reach 29%, more than EG.5 (21.7%).^52^ Hence, currently available vaccines may not address all challenges, and the SARS-CoV-2 variants could further diminish the effectiveness of these vaccines in the future.

### Current therapeutic drugs landscape

**Fig. 2:**
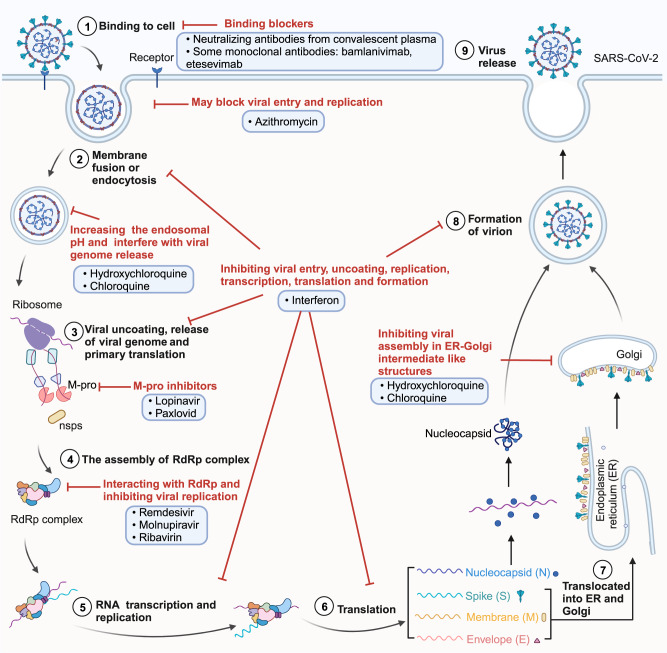
SARS-CoV-2 life cycle and the potential mechanisms of anti-SARS-CoV-2 therapeutics. (1) Binding to cell: the SARS-CoV-2 Spike protein recognizes and binds to the ACE2 receptor on host cells, initiating the process of cellular attachment. This step can be inhibited by neutralizing antibodies from convalescent plasma and monoclonal antibodies; (2) Fusion or endocytosis: subsequent to attachment, viral fusion or endocytosis with the host cell membrane ensues. Azithromycin, Hydroxychloroquine, and Chloroquine possess the capacity to modulate this crucial process; (3) Uncoating and genome release: viral uncoating follows, leading to the release of the viral genome and initiation of primary translation. M-pro inhibitors, like Lopinavir and Paxlovid, are tailored to impede this specific stage; (4) RdRp complex assembly: drugs such as Remdesivir, Molnupiravir, and Ribavirin specifically target the assembly process; (5) Viral RNA transcription and replication; (6) Translation of viral mRNA: viral mRNA translates into Nucleocapsid (N) and structural proteins (S, M, and E proteins); (7) Translocated into ER and Golgi: structural proteins are subsequently translocated into the ER and Golgi for maturation. Hydroxychloroquine and Chloroquine can block this process. (8) Formation of Virions: structural proteins combine with the nucleocapsid; (9) Virus release. Notably, interferons exert regulatory effects at multiple stages of the viral life cycle

Therapeutic drugs for COVID-19 can be mainly categorized into antiviral and immunomodulatory drugs (Table 3).^53–72^ The mechanisms of anti-SARS-CoV-2 therapeutics are outlined in Fig. 2. Antiviral drugs encompass nucleoside analogs, small molecule-based inhibitors, and antimalarials. Remdesivir, Molnupiravir, and Ribavirin are nucleoside analogs that can interact with the RNA-dependent RNA polymerase (RdRp) of SARS-CoV-2 to inhibit viral replication.^73–75^ Remdesivir is the initial drug approved by the FDA for treating COVID-19 via intravenous injection.^53^ Clinical studies have demonstrated that Remdesivir significantly improved clinical outcomes and expedited recovery time in patients with mild to severe COVID-19.^76^ Nevertheless, due to the requirement for intravenous administration and limited efficacy in critical COVID-19 cases, Remdesivir is only recommended for specific patients in particular medical settings. On November 4, 2021, Molnupiravir became the first oral antiviral drug approved in the UK to treat COVID-19 patients.^57^ The clinical trial proved that Molnupiravir had the potential to lower the risk of hospitalization and mortality.^77^ However, it is important to note that Molnupiravir has the potential to impact bone and cartilage growth, making it unsuitable for patients under 18 years old. Moreover, it may pose risks to fetal development, and its administration is not recommended during pregnancy.^78^ Ribavirin can cause a decrease in hemoglobin concentration, which may have adverse effects on COVID-19 patients.^79^

**Table 3:** The summary of authorized or approved COVID-19 therapeutics

Drug type	Drug name	Manufacture	Mechanism	Delivery route and dose	Recommended population	Approval status	Reference
**Antiviral drugs**	Remdesivir (Veklury)	Gilead Sciences	Inhibition of RdRp replication	Intravenous infusion; 200 mg on 1 d, then 100 mg from 2d (3 days for outpatients^a^, 5-10 days for inpatients).	Adult and pediatric patients (≥28 d and weighing ≥3kg): inpatients or nonhospitalized patients^a^	Approved by the FDA on October 22, 2020; Authorized EUA in many other countries.	^1,2^
	Favipiravir	Hisunpharm	Inhibition of RdRp replication	Oral; 1600 mg twice daily on 1d followed by 600 mg twice daily for 6-9 days.	Mild to moderate COVID-19 adult patients (prohibited for women who are known or suspected to be pregnant.)	Conditionally approved in China on February 15, 2020.	^3^
	AT-527 (Bemnifosbuvir)	Atea	Inhibition of RdRp replication	Oral.	-	Phase III.	^4^
	Molnupiravir (Lagevrio)	Merck	Inhibition of RdRp replication	Oral; 800 mg twice daily for 5 days.	Mild to moderate COVID-19 adult patients^a^	Approval on 4 November 2021 in the UK; Authorized EUA in many other countries.	^5–7^
	Paxlovid (nirmatrelvir plus ritonavir)	Pfizer	M-pro inhibitor	Oral; 300 mg nirmatrelvir with 100 mg ritonavir twice daily for 5 days.	Adult and pediatric patients^a^ (≥12 years and weighing ≥40 kg)	Approval on 31 December 2021 in the UK; Authorized EUA in many other countries.	^8–10^
	JT001 (VV116)	Junshi Biosciences Co., Ltd	Inhibition of RdRp replication	Oral; 0.6 g twice daily on 1d, then 0.3 g twice daily from 2 d to 5d.	Mild to moderate COVID-19 adult patients.	Approved in Uzbekistan; Conditionally approved in China on January 29, 2023.	^11–13^
	Ensitrelvir (Xocova)	Shionogi	M-pro inhibitor	Oral; 125 mg once daily for five days.	Mild to moderate COVID-19 adult patients.	Authorized EUA in Japan.	^14^
**immunomodulatory drugs**	Dexamethasone	Multiple	Corticosteroids	Oral or intravenous infusion; Dosage depends on the severity of the condition and response of the patient.	For patients with severe or critical COVID-19.	Approval in the UK; Authorized EUA in many other countries.	^15^
	Tocilizumab (Actemra)	Genentech	Anti IL-6 receptor	Intravenous infusion; Patients less than 30 kg weight: 12 mg/kg; Patients at or above 30 kg weight: 8 mg/kg; a second dose may be given at least 8 hours after the first dose. (max dose: 800 mg).	Hospitalized pediatric patients (≥2 years) who received systemic corticosteroids and require supplemental oxygen, non-invasive or invasive mechanical ventilation, or ECMO.	Approved by the FDA on 21 December 2022; Authorized EUA in many other countries.	^16,17^
	Anakinra (Kineret)	Sobi	IL-1 receptor antagonist	Subcutaneous injection; 100 mg daily for 10 days (100 mg every other day for a total of 5 doses over 10 days in patients who have severe renal insufficiency or end-stage renal disease).	Hospitalized adults requiring supplemental oxygen who are at risk of progressing to severe respiratory failure.	Authorized EUA on 8 November 2022 in the US.	^16,18^
	Baricitinib (Olumiant)	Eli Lilly	Janus kinase inhibitor	Oral; Pediatric patients (≥9 years): 4 mg/d once daily; Pediatric patients (≥9 years and ≥2 years): 2 mg/d once daily for 14 d.	Pediatric patients (≥2 years) requiring supplemental oxygen, invasive mechanical ventilation, or ECMO.	Approved by the FDA on 10 May 2022; Authorized EUA in many other countries.	^16,19^
	Vilobelimab(Gohibic)	InflaRx GmbH	Anti-C5a inhibitors	Intravenous infusion; 800 mg once, for a maximum of 6 doses.	Hospitalized adults who received IMV or ECMO within 48 h.	Authorized EUA on 12 April 2022 in the US.	^16,20^
	Monoclonal antibody and antibody cocktails	Multiple	Targeting the spike protein	Due to the ongoing mutation of the SARS-CoV-2 virus, monoclonal antibody and antibody cocktails such as Bamlanivimab, Sotrovimab, Etesevimab, REGEN-COV and Evusheld are no longer authorized for the treatment of COVID-19.			
	Convalescent plasma	Not recommended					

*EUA* emergency use authorization, *ECMO* extracorporeal membrane oxygenation, *IMV* invasive mechanical ventilation

^a^Patients have mild-to-moderate COVID-19, and are at high risk for progression to severe COVID-19, including hospitalization or death

Lopinavir and Paxlovid are small molecule-based inhibitors for COVID-19 treatment. Although Lopinavir exhibits some antiviral activity, its association with hepatotoxicity must also be considered.^80^ Paxlovid, targeting the M-pro in the SARS-CoV-2 genome, is highly recommended for COVID-19 treatment due to its sustained antiviral efficacy against emerging Omicron subvariants.^78,81^ Administering Paxlovid during the early stages of infection can significantly reduce hospitalization rates by 89% in high-risk patients.^82^ However, limitations of Paxlovid include a narrow treatment window and unsuitability for pregnant women, children under the age of 12, and patients with severe renal or hepatic impairment.^83,84^ Paxlovid can also induce extensive drug interactions, necessitating the verification of the patient’s daily medication to adjust the treatment approach of Paxlovid.^85^ Moreover, the extensive utilization of Paxlovid, coupled with amino acid substitutions in the M-protein vicinity, raises substantial concerns regarding potential resistance to Paxlovid. The swift upsurge in Paxlovid prescriptions may exert selective pressure on the virus, potentially driving its evolution toward resistance against this therapy. Concurrently, several research studies have pinpointed putative mutation sites associated with viral resistance to the drug. Mutations such as L50F, E166A/V, and L167F have been found to undermine the binding affinity between Paxlovid and M-protein, consequently diminishing Paxlovid’s efficacy against various SARS-CoV-2 variants. In contrast, the E166A/V mutation has been linked to a heightened resistance level.^86–88^ Importantly, the high cost of Paxlovid presents a significant obstacle for low-income nations, resulting in unequal access to treatment.^82,89^ Hydroxychloroquine, an antimalarial drug, has shown inhibitory effects against SARS-CoV-2 in vitro but lacks antiviral effects in vivo.^90,91^ It may lead to diarrhea and cardiomyopathy.^91,92^ Therefore, Hydroxychloroquine is not advisable for COVID-19 treatment.^81^

Immune modulators encompass convalescent plasma, antibiotics (such as Azithromycin), and various monoclonal antibodies. Convalescent plasma, containing polyclonal antibodies, can neutralize the virus and prevent infection. However, its use is constrained by varying antibody levels in individuals, transfusion-related risks, limited availability, and lack of quality standards. Consequently, convalescent plasma is only recommended for research purposes. Azithromycin has been shown to reduce viral replication.^93–95^ But it is associated with various side effects, particularly gastrointestinal and cardiovascular-related adverse events, leading to its exclusion from official COVID-19 treatment guidelines.^96^ The development of monoclonal antibodies is crucial in COVID-19 treatment. Monoclonal antibodies targeting the spike protein can bind to RBD or other regions, preventing viral entry into host cells. Multiple monoclonal antibodies and antibody cocktails have received emergency use authorization (EUA), demonstrating effectiveness in reducing hospitalization rates, mortality rates, and viral load.^97–102^ However, the emergence of prevalent variants with numerous RBD mutations has profoundly affected the therapeutic landscape of monoclonal antibodies. Many monoclonal antibodies and antibody combinations have lost their neutralizing efficacy against Omicron descendants.^7,103–106^

## Next-generation vaccines and therapeutics

**Fig. 3:**
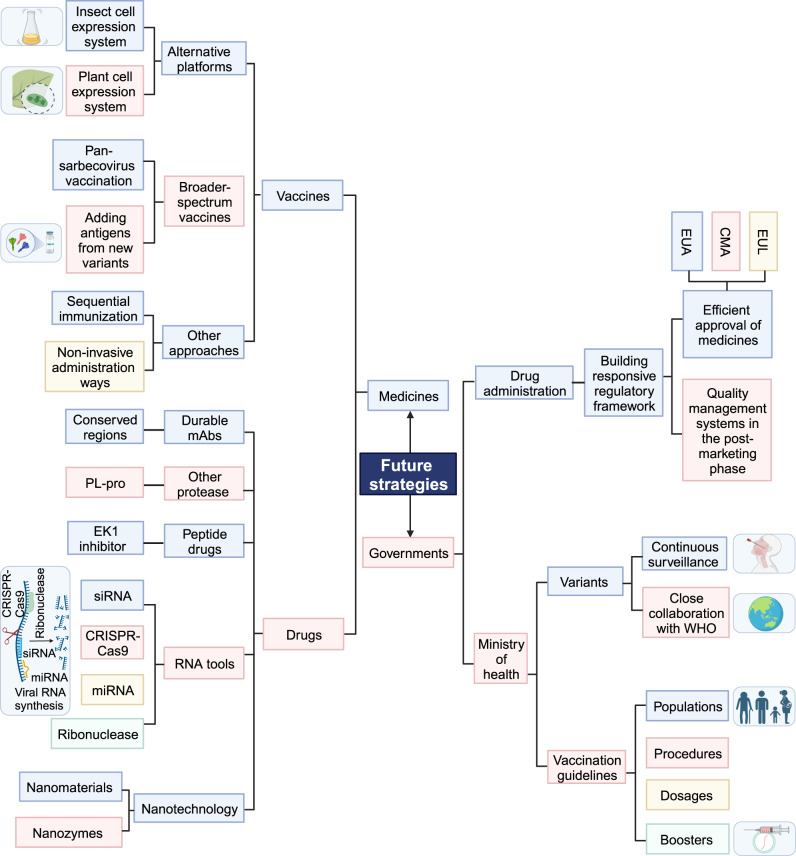
Potential intervention strategies for the future to optimize the management and prevention of SARS-CoV-2 infection. The strategies range from the development of future vaccines and drugs to the implementation of responsive measures by government agencies. EUA emergency use authorization, CMA conditional marketing authorization, EUL emergency use list program

Given the limited efficacy of current vaccines and drugs against emerging variants, there is a pressing requirement to advance next-generation vaccines and therapeutics (Fig. 3). In response to this challenge, the US government has pledged to invest 5 billion in novel COVID-19 vaccines and drugs.^107^

It may be time-consuming and costly to constantly update the vaccine according to the sequence of new variants, so a broad-spectrum vaccine will inevitably be developed to control the COVID-19 pandemic in the future. Developing a broad-spectrum vaccine requires the identification of antigens that elicit a wide range of antibodies to neutralize multiple SARS-CoV-2 variants. Beyond utilizing the chimeric S protein or RBD domains of circulating variants, it can also be designed based on the domain predicted by the model and algorithm. A study calculated the frequencies of mutation sites to design a novel antigen Span that encompasses high-frequency mutations.^108^ This approach not only can offer broad-spectrum protection targeting current strains but also has the potential to cover future mutant strains. Furthermore, Pan-sarbecovirus vaccination, incorporating antigens from new variants, may address the challenge of continuous virus mutation. A Pan-sarbecovirus vaccine, Mosaic-8b, incorporates RBDs from SARS-CoV-2 and seven animal coronaviruses, inducing broader neutralizing antibodies in mice and nonhuman primates.^109^

Classifying SARS-CoV-2 into distinct serotypes also helps to guide the selection of variants to be included in updated broader-spectrum vaccines. According to the analysis of Etienne Simon-Loriere and Olivier Schwartz, compared with Alpha/Beta/Gamma/Delta, Omicron variants (BA.1/BA.2/BA.3) exhibit limited cross-neutralization and a greater phylogenetic distance.^110^ They advocate for designating Omicron as a distinct SARS-CoV-2 serotype 2 while categorizing the wild-type virus and other VOCs as serotype 1. Recent research supports this classification and revealed that XBB and BQ.1 variants exhibit more significant antigenic drift than other Omicron variants.^111^ Developing new vaccines to incorporate information regarding XBB variants is imperative. Hence, identifying the serotypes of viral variants assists scientists in pinpointing antigens for next-generation polyvalent vaccines and evaluating their potential for integration with existing vaccines.

So far, the vast majority of approved COVID-19 vaccines are administered by intramuscular injection. Some other non-invasive administration ways might be considered when developing new vaccines in the future (Fig. 3). For instance, vaccination via inhalation or oral administration is more friendly and acceptable to the elderly and children.^112–115^ Moreover, the self-administration of such non-invasive vaccines could be feasible, which helps the quick immunization of large populations, especially when encountering pandemics. Heterologous immunization is also recommended to optimize the efficacy of new vaccines. Many research studies have shown that heterologous immunizations can confer cross-protection against various variants.^116–118^ Therefore, we recommend utilizing vaccines from different platforms as the primary choice for boosters.

Regarding next-generation therapeutics, the development of drugs targeting RdRp and M-pro remains a viable and sensible approach.^119^ These two targets have shown fewer observed mutations and have demonstrated effectiveness against all existing variants. The 5 billion investment plan emphasized the importance of developing more durable monoclonal antibodies against new variants. Exploring pan-coronavirus antibodies should target more conserved regions in spike protein, such as the NTD and the SD1 domain in the S1, the stem helix region and FP regions in the S2, and some RBD class 3 and 4 antibodies.^64,120,121^ While they can broadly inhibit infection and mitigate the severity of COVID-19, their neutralizing activity may be limited. Combinations of these mAbs or pairing them with other potent mAbs may be a feasible strategy against COVID-19 by improving the synergistic effect between antibodies and reducing the risk of drug resistance.

Additionally, the development of peptide-based pan-coronavirus inhibitors represents a promising therapeutic avenue. These inhibitors offer several advantages against SARS-CoV-2 infection. Peptide drugs are known for their high specificity and excellent tolerability, with the potential to extend their half-life through modifications.^122,123^ Moreover, peptide drugs are cost-effective to synthesize and exhibit stability, allowing for room-temperature storage and transportation.^124^ Their low molecular weight also facilitates convenient administration in inhaled or oral forms.^125^ Notably, peptides can be rapidly created and adjusted in silico techniques, which is crucial for expedited drug screening in the future.^126^ Some studies have demonstrated the effectiveness of peptides EK1 and EK1C4 targeting the HR1 domain in inhibiting SARS-CoV-2 infection.^127–130^ These two inhibitors even retain their strong efficacy in blocking XBB.1.5 infection.^130^ Therefore, the HR1 domain emerges as a pivotal target for the development of pan-coronavirus drugs, with the potential to serve as a broad-spectrum inhibitor for Omicron and future coronaviruses.

Other potential therapeutics involve new protease inhibitors, antiviral tools targeting viral RNA (siRNA, miRNA, CRISPR-Cas9 system, and ribonuclease), and nanotechnology (Fig. 3).^39,131^ Small molecule-based inhibitors papain-like protease (PL-pro) instead of M-pro can also target other vital proteases in virus replication.^132,133^ siRNA acts directly and specifically on viral RNA, reducing the risk of drug resistance and improving drug safety.^134–136^ Nanomaterials can be engineered to target specific cells, reducing drug toxicity.^137^ Some nanozymes exhibit favorable biological distribution and can inhibit virus infection without harming host cells, thereby optimizing therapeutic outcomes and reducing drug side effects.^138,139^ Finally, aiming for convenient treatment, future therapeutic drugs will likely focus on oral administration. As a novel oral nucleotide analog drug, VV116 has recently received conditional approval for marketing in China.^140^

## Approval of medicines and government guidelines

In usual circumstances, the development of drugs and vaccines entails rigorous testing and extensive clinical trials. This process often spans several years before obtaining marketing approval. However, due to the highly infectious SARS-CoV-2 and its severe effects on human health, traditional drug regulatory and approval processes are no longer suitable. Many countries implemented emergency authorizations for COVID-19 medicines to curtail viral transmission. In the United States, the FDA may issue an EUA after the HHS Secretary declares the existence of circumstances justifying such authorization and consulting with relevant authorities.^141^ In the European Union, member states grant conditional marketing authorization (CMA) to COVID-19 medicines through EMA. Some conditions must be met to obtain CMA: the anticipated benefits of the medicine outweigh its potential risks; pharmaceutical companies must submit further clinical trials and additional data to evaluate the safety and efficacy of the drugs or vaccines.^142^ In China, vaccines that respond to major public health emergencies may be granted conditional approval or permission for emergency use. WHO uses the Emergency Use List program (EUL) to evaluate and list unlicensed vaccines, therapeutics, and in vitro diagnostics.^143^ Emergency authorization policies differ across regions, each with its unique characteristics. When comparing the United States and the European Union to the Chinese government, there are variations in the scope and maturity of their emergency authorization systems. Currently, emergency authorization in China primarily covers vaccines, lacking comprehensive regulations for therapeutic drugs. Contrasted with the United States empowering the FDA directly and the United Kingdom making swift decisions through legislative means, the EU’s review process (at least 70 days) seems more intricate, time-consuming, and relatively cautious. However, it emphasizes transparency and frequent public disclosure of information. Although the COVID-19 pandemic is no longer classified as a Public Health Emergency of International Concern, the emergence of new variants may render numerous drugs and vaccines ineffective. Hence, drug authorities should remain vigilant in the global strain tracking by WHO and establish an expeditious approval process for new drugs and vaccines targeting threatening variants. For instance, facing the raging XBB variant, China first authorized the emergency use of a recombinant trivalent XBB protein vaccine produced by WESTVAC BIOPHARMA.^144^ Targeting the spike protein of the XBB.1.5 and other variants, this vaccine can self-assemble into stable trimeric protein particles and induce high levels of neutralizing antibodies against XBB.1.5, XBB.1.16, XBB.1.9.1, and EG.5. Subsequently, the FDA granted EUA for updated COVID-19 vaccines developed by Pfizer, Moderna, and Novavax, which include the XBB.1.5 antigen in their new formulations.^145^ In summary, the efficacy of emergency authorization policies was evident in supervising vaccines and drugs during the pandemic. To capitalize on past successes and ensure future preparedness, regulatory authorities across nations should derive valuable lessons from the challenges posed by the COVID-19 crisis. In the post-pandemic era, regulatory authorities can respond more flexibly, rapidly, and efficiently to potential future public health emergencies by developing and improving relevant regulations and optimizing procedures of authorization and technical reserves (Fig. 3). Meanwhile, the post-marketing phases need to prioritize the establishment and enhancement of quality management systems to further validate the safety and efficacy of vaccines and drugs. Strategic leveraging of past experiences and ongoing improvements will contribute to a more logical and practical regulatory framework for the oversight of vaccines and drugs (Fig. 3).

Health agencies should formulate future vaccination guidelines, particularly targeting different populations. Several countries have implemented vaccination strategies, providing specific recommendations for vaccination procedures and dosages for primary and booster vaccination. In Germany, STIKO does not recommend injecting COVID-19 vaccines for healthy infants, children, and adolescents. It suggests that individuals aged 18 and above should receive three antigen exposures to acquire fundamental immunity, including at least two vaccine doses.^146^ STIKO also suggests that immunocompromised patients and their close contacts, people over 60 years old, individuals over 6 months old with relevant underlying conditions, and people at high risk of infection should receive a booster vaccination in autumn or one year after their last antigen exposure.^146,147^ Timely vaccination against COVID-19 is also advised for breastfeeding or second-trimester pregnant women who have not been vaccinated.^148^ The Ministry of Health in Singapore recommends primary vaccination for those aged 6 months to 4 years and booster doses for those aged 5 years and above. Furthermore, people aged 60 and above, residents of aged care facilities, and medically vulnerable individuals aged 12 years and above are advised to receive another booster dose one year after their initial booster.^149^ In the United Kingdom, the recommendation is for individuals aged 6 months and older to receive COVID-19 vaccinations. In the autumn of 2023, boosters will be administered to high-risk vulnerable populations, including individuals aged over 65, residents of nursing homes, healthcare professionals, and others.^150^ CDC emphasizes that individuals over 6 months should receive the latest vaccine as part of their initial immunization or as a booster.^151^ However, many countries do not have a clearly defined vaccination strategy, and globally harmonized vaccination recommendations are still lacking. In the effort to combat the ongoing pandemic, it is paramount that health agencies across various nations maintain vigilant surveillance of SARS-CoV-2 variants, collaborate in information-sharing, judiciously select the appropriate antigens for the new vaccines before autumn, and design better vaccination strategies for vulnerable populations (Fig. 3). Boosters can provide benefits across various age groups and help reduce the risk of virus transmission. Therefore, it is also advisable to offer boosters as an option for individuals in low-risk groups. However, considering the financial and human resource constraints that some countries may face, large-scale free booster vaccination programs might not be feasible. In addition to ensuring free universal immunization, health departments should proactively identify priority groups for booster and cover the costs. Finally, beyond the development of vaccination guidelines, Health agencies should continue to conduct public health education and disseminate information on scientific epidemic prevention. This not only supports the work of health departments but also enhances public health awareness, reducing the probability of COVID-19 or other disease infections.

## Conclusions

Given the seriousness and emergency nature of COVID-19, scientists have rapidly developed numerous vaccines and drugs to control virus transmission. Drug regulatory authorities have also promptly adjusted policies and granted emergency use authorization for some vaccines and drugs to expedite the deployment of medicines. As a result, over the past years, vaccines and drugs have helped us to make significant progress in combating this pandemic. However, the virus continues to mutate, causing persistent infections and deaths and a decline in the effectiveness of early vaccines and drugs.

In the future, the global community must constantly monitor emerging variants and collaborate closely to share relevant information. This proactive approach would enable the timely detection of variants that may trigger waves of infections and facilitate the execution of suitable prevention and control. Additionally, exploring alternative development platforms, updating antigens, investigating broad-spectrum medicines, and improving delivery methods should be considered to enhance vaccine and drug preparedness during pandemics. Achieving these objectives requires relevant policy support like EUA from the drug administration. Furthermore, the drug administration should assist health management departments in optimizing future vaccination strategies, including determining suitable populations, appropriate dosages, and dosing intervals, thereby maximizing vaccine efficacy.

## Data Availability

The data included in this study are available upon request from the corresponding author.
